# The Cholinergic Drug Galantamine Alleviates Oxidative Stress Alongside Anti-inflammatory and Cardio-Metabolic Effects in Subjects With the Metabolic Syndrome in a Randomized Trial

**DOI:** 10.3389/fimmu.2021.613979

**Published:** 2021-03-11

**Authors:** Carine Teles Sangaleti, Keyla Yukari Katayama, Kátia De Angelis, Tércio Lemos de Moraes, Amanda Aparecida Araújo, Heno F. Lopes, Cleber Camacho, Luiz Aparecido Bortolotto, Lisete Compagno Michelini, Maria Cláudia Irigoyen, Peder S. Olofsson, Douglas P. Barnaby, Kevin J. Tracey, Valentin A. Pavlov, Fernanda Marciano Consolim Colombo

**Affiliations:** ^1^Hypertension Unit, University of São Paulo (USP), São Paulo, Brazil; ^2^Postgraduate Program in Health Science, Midwestern State University (UNICENTRO), Paraná, Brazil; ^3^Nursing Department Graduate Program in Nanosciences and Biosciences, Nove de Julho University (UNINOVE), São Paulo, Brazil; ^4^Department of Physiology, Federal University of São Paulo (UNIFESP), São Paulo, Brazil; ^5^Biomedical Sciences Institute Department of Physiology and Biophysics, University of São Paulo (USP), São Paulo, Brazil; ^6^Laboratory of Immunobiology, Department of Medicine, Center for Bioelectronic Medicine, Karolinska Institutet, Stockholm, Sweden; ^7^The Feinstein Institutes for Medical Research, Northwell Health, Manhasset, NY, United States

**Keywords:** metabolic syndrome, galantamine, cholinergic, oxidative stress, heart rate variability, autonomic modulation, inflammation

## Abstract

**Background:** The metabolic syndrome (MetS) is an obesity-associated disorder of pandemic proportions and limited treatment options. Oxidative stress, low-grade inflammation and altered neural autonomic regulation, are important components and drivers of pathogenesis. Galantamine, an acetylcholinesterase inhibitor and a cholinergic drug that is clinically-approved (for Alzheimer's disease) has been implicated in neural cholinergic regulation of inflammation in several conditions characterized with immune and metabolic derangements. Here we examined the effects of galantamine on oxidative stress in parallel with inflammatory and cardio-metabolic parameters in subjects with MetS.

**Trial Design and Methods:** The effects of galantamine treatment, 8 mg daily for 4 weeks or placebo, followed by 16 mg daily for 8 weeks or placebo were studied in randomly assigned subjects with MetS (*n* = 22 per group) of both genders. Oxidative stress, including superoxide dismutase (SOD), catalase (CAT), and glutathione peroxidase activities, lipid and protein peroxidation, and nitrite levels were analyzed before and at the end of the treatment. In addition, plasma cytokine and adipokine levels, insulin resistance (HOMA-IR) and other relevant cardio-metabolic indices were analyzed. Autonomic regulation was also examined by heart rate variability (HRV) before treatment, and at every 4 weeks of treatment.

**Results:** Galantamine treatment significantly increased antioxidant enzyme activities, including SOD [+1.65 USOD/mg protein, [95% CI 0.39–2.92], *P* = 0.004] and CAT [+0.93 nmol/mg, [95% CI 0.34–1.51], *P* = 0.01], decreased lipid peroxidation [thiobarbituric acid reactive substances [log scale 0.72 pmol/mg, [95% CI 0.46–1.07], *P* = 0.05], and systemic nitrite levels [log scale 0.83 μmol/mg protein, [95% CI 0.57–1.20], *P* = 0.04] compared with placebo. In addition, galantamine significantly alleviated the inflammatory state and insulin resistance, and decreased the low frequency/high frequency ratio of HRV, following 8 and 12 weeks of drug treatment.

**Conclusion:** Low-dose galantamine alleviates oxidative stress, alongside beneficial anti-inflammatory, and metabolic effects, and modulates neural autonomic regulation in subjects with MetS. These findings are of considerable interest for further studies with the cholinergic drug galantamine to ameliorate MetS.

## Introduction

The metabolic syndrome (MetS), comprising a combination of central (abdominal) obesity, dyslipidemia, and elevated fasting glucose and blood pressure is a disorder of pandemic proportions (1). MetS is linked to a significantly increased risk for type 2 diabetes, cardiovascular disease, cancer, and other debilitating and lethal diseases (1, 2). Despite the enormous and growing deleterious impact of MetS on our modern society and healthcare, treating this disorder as a whole has been challenging and remains inefficient (3). Low-grade chronic inflammation and increased oxidative stress have been identified as substantial contributing factors and drivers of MetS pathobiology (2, 4–7). In addition, autonomic nervous system dysfunction, with increased sympathetic and decreased vagus nerve activities, has been documented in MetS patients (8–12) and related to arterial hypertension, diabetes, heart failure, and stroke (13, 14). Accordingly, lowering chronic inflammation and oxidative stress, and improvement of autonomic nervous system function are promising therapeutic approaches in MetS (3, 6, 10, 12, 15–18).

In addition to its “classical” physiological functions in neural autonomic regulation, the vagus nerve controls inflammation through a brain-integrated physiological mechanism termed *the inflammatory reflex* (19, 20). Experimental evidence indicates that activation of brain cholinergic signaling by galantamine and other cholinergic compounds is functionally integrated with efferent vagus nerve activity within the inflammatory reflex in the regulation of peripheral inflammation (20–25). Galantamine is an inhibitor of the enzymatic activity of acetylcholinesterase (AChE), which degrades acetylcholine into choline and acetate, and a clinically-approved cholinergic drug for Alzheimer's disease (26–29). In preclinical settings, galantamine alleviates inflammation and metabolic derangements in murine models of many diseases, including high-fat diet-induced obesity and MetS (23, 30–33). In addition, galantamine alters autonomic nervous system modulation toward parasympathetic (vagus nerve) predominance (32, 34). Recently, we demonstrated that 12 weeks of administration of low to moderate galantamine doses (which are clinically approved), alleviated systemic inflammation and insulin resistance, and altered autonomic regulation in patients with MetS (35).

In addition to inflammation, oxidative stress is an important contributor to MetS pathogenesis (4, 6, 15). A prolonged state of oxidative stress is manifested by reduced activities of antioxidant enzymes and increased lipid peroxidation (4, 5, 36). The redox system imbalance and oxidative stress in MetS are associated with pro-inflammatory and pro-thrombotic pathways that drive insulin resistance and cardio-metabolic derangements, which negatively affect the clustering components of MetS, promote atherogenesis and increase the risks of type 2 diabetes and cardiovascular disease (15, 36, 37). Considering the importance of the interplay between inflammation and oxidative stress in the pathogenesis of many disorders, including MetS (16, 38, 39) and previous work indicating the anti-inflammatory efficacy of galantamine, we hypothesized that this cholinergic drug will alleviate oxidative stress indices in patients with MetS. We examined the effects of galantamine treatment for 12 weeks on superoxide dismutase (SOD), catalase (CAT), and glutathione peroxidase (GPx) activities, lipid and protein oxidation, and nitrite levels in MetS patients. In parallel, we reexamined the anti-inflammatory, and other galantamine effects, and provided insight into the time course of galantamine autonomic modulation by assessing heart rate variability (HRV) components.

## Methods

### Study Design

The present study is based on analysis of data obtained from a prospective, randomized, double-blind, placebo-controlled trial with galantamine treatment in patients with the MetS. The study protocol was reviewed and approved by the Institutional Ethical Committee and the Human Subject Protection Committee of Heart Institute (InCor) and Clinic Hospital of the University of São Paulo, Brazil (number 11672/555738), and registered at ClinicalTrials.gov (NCT02283242). It was conducted following the World Medical Association International Code of Medical Ethics (Declaration of Helsinki, 1964; revised in 2008).

The complete study protocol and some results indicating galantamine effects on inflammation, HRV and cardio-metabolic parameters were recently published (35). The complete trial consort flow diagram also was previously published (35); it is provided here as Supplementary Figure 1. Participants in the study were of both genders, aged 18–59 years. They were diagnosed with MetS according to the ATP III criteria consisting of the presence of at least three of the following five parameters: increased abdominal circumference (≥102 cm for men and ≥88 cm for women); low plasma HDL cholesterol levels (<40 mg/dl for men and <50 mg/dl for women); increased values for plasma triglycerides (≥150 mg/dl); elevated blood pressure (≥130 mmHg systolic blood pressure or ≥85 mm Hg diastolic blood pressure), and increased plasma glucose levels (≥100 mg/dl). The following exclusion criteria were applied: symptoms and signs of cardiovascular disease or previous diagnosis of cardiac arrhythmias; coronary artery disease; valvular disease; heart failure; chronic obstructive pulmonary disease; chronic inflammatory diseases; cancer; positive status for HIV; abuse of alcohol or other illicit substances in the months prior to study entry; chronic use of medications, including drugs that have known or probable interaction with galantamine (serotonin reuptake inhibitors, amitriptyline, fluoxetine, fluvoxamine, ketoconazole, oxybutynin, paroxetine, quinidine); symptoms and signs of neurologic and autonomic diseases; past history of major depression, suicidal ideation, and history of eating disorders; triglyceride levels 400 mg/dl or higher; known history of liver disease or levels of aspartate transaminase (AST) or alanine transaminase (ALT) 200 U/l or higher; office BP 160 mmHg or higher or DBP 110 mmHg or higher; and abnormal renal and thyroid function. After the initial screening and consideration of inclusion and exclusion criteria, 60 eligible subjects of both genders were consecutively randomized in a 1:1 ratio to treatment with galantamine or placebo for 12 weeks. In order to reduce sex bias, subjects were stratified by sexual phenotype prior to being randomized to the galantamine or placebo arm. Recruitment of study participants took place until 30 men and 30 women were recruited to allow randomization in equally divided groups by sex. Study participants were randomly assigned at a 1:1 ratio as follows: 30 subjects (15 males and 15 females) were assigned to galantamine treatment and 30 subjects (15 males and 15 females) to placebo. A computer-generated random sequence method was used to allocate the participants to the corresponding groups. Randomized packages of placebo and active drug were numbered from 1 to 60 (http://graphpad.com/quickcalcs/randomize1/). Central Pharmacy staff members who did not participate in the study conducted the randomization and were responsible for the drug delivery and inventory. Specific drug accountability included quantities dispensed/received (capsule counting) every month, serial numbers, expiration date, and drug code number. To achieve masking (blinding), galantamine capsules were reencapsulated at the Central Pharmacy of the Clinical Hospital, Medical School of University of São Paulo. Active drug and placebo capsules were presented in identical medication packaging. The study drug packages were stored in a secure, temperature-controlled medication room according to standard operating procedures. Subjects received packages of study drug for 30 ± 3 days, every 4 weeks for a total of 12 weeks. Study participants, investigators, and outcome assessors were blinded to the group assignment.

After reencapsulation, galantamine hydrobromide extended-release capsules of 8 mg, commercially available as Reminyl^®^ ER (manufactured by Janssen-Cilag Pharmaceuticals, Johnson & Johnson, Brazil), were administered in a dose of one tablet per day (8 mg/days) for 4 weeks and then titrated to two tablets per day (16 mg/days) for 8 weeks. At each medical visit, the exact number of galantamine or placebo capsules were given to the patients. Pill counting in the next visit confirmed the adherence to the treatment. All patients with some exceptions such as a few patients who did not come for fat depot tissue assessment as previously noted (35) completed the study. In the course of the study, 44 (22 per arm) out of 60 patients had the Finometer determination of blood pressure, which was used for HRV analysis, recorded at all four key time points of the study. These were: T0–basal measurements before treatment; T1–following 4 weeks of galantamine 8 mg/days or placebo; T2–following 4 weeks of galantamine 16 mg/days or placebo; and T3–after 4 final weeks of galantamine 16 mg/days or placebo. CONSORT 2010 guidelines (http://www.consort-statement.org) were followed during the preparation of this manuscript.

### Determination of Oxidative and Nitrosative Stress, Metabolic Profile, and Inflammatory Mediators

Venous blood samples were withdrawn twice, at baseline (T0) and the end of the protocol (T3). Parameters of oxidative stress were determined in plasma as previously described (13). Quantification of superoxide dismutase (SOD) activity was performed based on the inhibition of the reaction between O_2_ and pyrogallol (40). Catalase (CAT) activity was determined by measuring the reduction in H_2_O_2_ absorbance at 240 nm (41, 42). Glutathione peroxidase (GPx) activity was based on the consumption of NADPH (22). For the TBARS assay (lipid peroxidation), trichloroacetic acid (10%, w/v) was added to the homogenate for proteins precipitation and sample acidification (43). The mixture was centrifuged (3,000 g, 3 min), the protein-free sample extracted and thiobarbituric acid (0.67%, w/v) was then added to the reaction medium and tubes were placed in a water bath (100 °C) for 15 min. Absorbance was measured at 535 nm using a spectrophotometer (43). The protein oxidation was measured by a reaction of protein carbonyl groups with 2,4-dinitrophenylhydrazine to form 2,4-dinitrophenylhydrazone, which can be quantified spectrophotometrically. The reaction product was measured at 360 nm (43). The Griess reagent was used to determine the plasma nitrites (NO^−2^) (13). Blood tests, including triglycerides, total, HDL and LDL cholesterol as well as fasting glucose, were performed according to standard protocols. Plasma samples were stored at −80°C before analysis. Cytokines, adipokines and insulin were analyzed using multiplex immunoassay (all from Millipore): HCYTMAG-60K-PX41 for TNF; HADK2MAG-61K for leptin and insulin; and HADK1MAG-61K for adiponectin. Individual values for leptin and adiponectin were used to calculate the leptin/adiponectin ratio as previously described (35).

### Hemodynamic Parameters and Heart Rate Variability (HRV) Evaluation

All subjects were asked to abstain from exercise for 24 h prior to study visits and from drinking caffeinated products on the morning of their evaluation. During a 15 min study period, subjects were supine and awake in a quiet room while blood pressure waveforms (BP) were captured and stored using a digital photoplethysmograph device (Finometer, Finapres Medical System BV, Holland) as previously described (35). Recordings were visually inspected to remove non-stationary data and sequential pulse intervals (PI) and then used to compute HRV in both frequency and time domains as previously described in detail (35). All analyses adhered to standards developed by the Task Force of the European Society of Cardiology and the North American Society of Pacing and Electrophysiology (44).

### Statistical Analysis

As noted before (35), the proposed sample size for this trial was 60 subjects (*n* = 30 per group), based on resources available and in consistency with previous clinical studies (45, 46). Of note, a sample size of 30 in each group will have 86% power to detect an effect size of 0.8 using a 2-group *t*-test with a 0.05 2-sided significance level. This is consistent with the power analysis in a placebo-controlled study of the effects of a drug (rosiglitazone) once daily for 12 weeks on inflammatory and metabolic indices in 60 patients with MetS (45).

The Kolmogorov-Smirnov test was used to test for normality. Descriptive statistics were calculated separately for each of the two treatments (galantamine and placebo) using mean ± standard deviation and median (25th percentile, 75th percentile) for continuous data, and frequencies and percentages for categorical data. To establish baseline comparability of the two randomized treatment arms, the chi-square test was used to compare categorical variables (i.e., gender) and the Mann-Whitney test, the non-parametric counterpart to the two-sample *t*-test, was used to compare continuous measures (Table 1).

**Table 1:** Baseline characteristics.

	**Placebo group**			**Galantamine group**			
	***n***	**Mean**	**SD**	***n***	**Mean**	**SD**	***P*-value**
**AGE AND ANTROPOMETRY**							
Age (years)	22	42.05	8.67	22	40.55	7.86	0.8
Male/Female	22	12	10	22	9	13	0.5
Body mass index (kg/m^2^)	22	33.88	4.07	22	34.67	3.31	0.5
Waist circumference (cm)	22	108.59	9.25	22	108.73	8.08	0.9
**METABOLIC MARKERS**							
Glucose (mg/dl)	22	98.95	10.88	22	101.55	9.46	0.4
Insulin (mIU/L)	22	20.42	23.75	22	18.09	7.54	0.7
HOMA-IR	22	4.93	5.65	22	4.43	1.91	0.7
HDL cholesterol (mg/dl)	22	42.95	9.55	22	43.64	5.92	0.9
LDL cholesterol (mg/dl)	22	127.36	44.12	22	127.77	38.26	0.9
Triglycerides (mg/dl)	22	157.64	66.67	22	172.73	85.94	0.6
Total cholesterol (mg/dl)	22	198.36	48.63	22	200.14	37.92	0.9
**BLOOD PRESSURE, HEART RATE AND HRV**							
SBP (mmHg)	22	127.41	13.20	22	121.91	8.38	0.1
DBP (mmHg)	22	82.73	8.76	22	78.91	5.98	0.1
Heart rate (b/min)	22	68.88	10.04	22	72.02	9.89	0.3
LF (ms)	22	546.11	532.61	22	716.11	1390.88	0.6
HF (ms)	22	798.72	1476.86	22	575.77	945.50	0.6
LF (nu)	22	48.50	16.60	22	52.86	15.85	0.4
HF (nu)	22	50.77	16.13	22	47.14	15.86	0.5
LF/HF	22	1.35	1.00	22	1.67	1.12	0.3
**INFLAMMATORY MARKERS**							
TNF (pg/ml)	22	12.22	4.82	22	12.49	4.68	0.9
Leptin (ng/ml)	22	27.90	14.93	22	33.08	21.91	0.4
Adiponectin (μg/ml)	22	8.76	1.75	22	8.72	1.85	0.9
Leptin/adiponectin ratio	22	3.32	1.86	22	4.03	2.51	0.3
**OXIDATIVE STRESS MARKERS**							
SOD (USOD/mg prot)	16	3.26	1.21	12	3.68	1.88	0.5
CAT (nmol/mg)	21	2.63	0.74	21	2.67	0.61	0.9
GPx (nmol/min/mg prot)	22	14.42	8.20	20	20.20	16.86	0.2
TBARS (pmol/mg)	22	9.28	9.11	21	8.26	7.43	0.7
PCG (nmol/mg prot)	19	0.96	0.32	21	1.06	0.44	0.5
Nitrite (umol/mg prot)	22	0.27	0.18	21	0.41	0.37	0.1

*HRV, heart rate variability; LF, low frequency; HF, high frequency; SOD, superoxide dismutase; CAT, catalase; GPx, Glutatione peroxidase; TBARS, thiobarbituric acid reactive substances; PCG, protein carbonyl groups*.

Repeated measures analysis of variance (RMANOVA) with a mixed models approach was used to determine if the two groups behave differently over time (i.e., the group x time interaction) for each of the following measures: CAT, SOD, GPx, TBARS, carbonyls, nitrites, low frequency (LF) of HRV, high frequency (HF) of HRV (absolute and normalized values), LF/HF ratio, BMI, SBP, DBP, glucose, HDL cholesterol, LDL cholesterol, triglycerides, total cholesterol, TNF, leptin, adiponectin, and HOMA-IR. For all analyses, the standard assumptions of Gaussian residuals and equality of variance were tested. When normality assumption was not met, the logarithm transformation was used for the analysis of these variables. The repeated within-subjects factor was time (pre- and post), and the within-subjects factor was treatment group (galantamine and placebo). Data analyzed on the raw scale are reported as the arithmetic difference, calculated as post- minus pre-, and standard deviation (SD) for each group. “Positive” values indicate an increase in the measure from pre- to post- and “negative” values indicate decreases in the measure from pre- to post-. Those variables analyzed on the log scale were back transformed and summarized using the geometric mean ratio (GMR), which corresponds to the magnitude of difference between pre- and post- measurements; values >1 indicate an increase from pre- to post- and values <1 indicate a decrease from pre- to post-. These variables are summarized in their original units of measurement for the columns labeled “Baseline” and “Follow-up” in Tables 1–3. For the columns labeled “Treatment × Time Interaction Effect,” they are summarized in the log scale (Tables 2, 3).

**Table 2:** Effect of galantamine on oxidative stress parameters.

**Oxidative stress markers**	**Baseline**						**Follow-up**						**Treatment** **×** **Time interaction effect**			
	**Placebo group**			**Galantamine group**			**Placebo group**			**Galantamine group**						
	***n***	**Mean**	**SD**	***n***	**Mean**	**SD**	***n***	**Mean**	**SD**	***n***	**Mean**	**SD**	**Effect**	**95% CI**		***P*-value**
SOD (USOD/mg prot)	16	3.26	1.21	12	3.68	1.88	16	2.80	21.17	12	4.45	2.06	1.65	0.39	2.96	0.004
CAT (nmol/mg)	21	2.63	0.74	21	2.67	0.61	21	3.06	0.82	21	3.99	1.05	0.93	0.34	1.51	0.01
GPx (nmol/min/mg prot)	22	14.42	8.20	20	20.20	16.86	22	17.96	14.12	20	18.56	14.05	−1.64	−9.31	6.03	0.3
TBARS (pmol/mg)^*^	22	9.28	9.11	21	8.26	7.43	22	10.93	12.24	21	5.48	2.70	0.72^*^	0.46	1.07	0.05
PCG (nmol/mg prot)	19	0.96	0.32	21	1.06	0.44	19	0.91	0.22	21	0.83	0.20	−0.07	−0.21	0.06	0.2
Nitrite (umol/mg prot)^*^	22	0.27	0.18	21	0.41	0.37	22	0.35	0.32	21	0.29	0.19	0.83^*^	0.57	1.20	0.04

* *These variables were analyzed on the logarithmic scale. SOD, superoxide dismutase; CAT, catalase; GPx, glutatione peroxidase; TBARS, thiobarbituric acid reactive substances; PCG, protein carbonyl groups*.

**Table 3:** Effect of galantamine on metabolic, hemodynamic, and inflammatory markers.

	**Baseline**						**Follow -up**						**Treatment** **×** **Time interaction effect**			
	**Placebo group**			**Galantamine group**			**Placebo group**			**Galantamine group**			**Effect**	**95% CI**		***P*-value**
	***n***	**Mean**	**SD**	***n***	**Mean**	**SD**	***n***	**Mean**	**SD**	***n***	**Mean**	**SD**				
**ANTROPOMETRY**																
Body mass index (kg/m^2^)	22	33.88	4.07	22	34.67	3.31	22	33.65	4.39	22	34.31	3.63	0.66	−1.78	3.10	0.7
Waist circumference (cm)	22	108.59	9.25	22	108.73	8.08	22	106.82	9.26	22	106.00	8.69	−0.82	−6.28	4.65	0.3
**METABOLIC MARKERS**																
Glucose (mg/dl)	22	98.95	10.88	22	101.55	9.46	22	97.91	10.80	22	95.05	8.09	−2.87	−8.67	2.94	0.1
Insulin (mIU/L)^*^	22	20.42	23.75	22	18.09	7.54	22	25.29	27.35	21	11.75	6.90	0.60^*^	0.40	0.89	0.01
HOMA–IR^*^	22	4.93	5.65	22	4.43	1.91	22	3.69	2.21	21	2.85	2.09	0.51^*^	0.32	0.79	0.01
HDL cholesterol (mg/dl)	22	42.95	9.55	22	43.64	5.92	22	42.64	7.90	21	43.11	7.78	1.07	−3.75	5.91	0.7
LDL cholesterol (mg/dl)	22	127.36	44.12	22	127.77	38.26	22	117.45	36.65	21	114.30	31.40	−3.16	−24.22	17.9	0.5
Triglycerides (mg/dl)	22	157.64	66.67	22	172.73	85.94	22	146.31	74.20	21	150.24	77.08	3.92	−42.67	50.51	0.6
Total cholesterol (mg/dl)	22	198.36	48.63	22	200.14	37.92	22	188.59	38.14	21	183.10	36.35	−5.49	−28.46	17.47	0.3
**BLOOD PRESSURE, HEART RATE, AND HRV**																
SBP (mmHg)	22	127.41	13.2	22	121.91	8.38	22	124.55	14.34	22	123.50	11.55	−1.04	−8.97	6.87	0.2
DBP (mmHg)	22	82.73	8.76	22	78.91	5.98	22	76.68	8.53	22	76.36	10.10	−0.31	−6.00	5.37	0.2
Heart rate (b/min)	22	68.88	10.04	22	72.02	9.89	22	69.01	8.24	22	72.01	9.59	2.92	−2.89	8.74	0.9
LF (ms)^*^	22	546.11	532.61	22	716.11	1390.88	22	497.28	291.15	22	329.83	253.07	0.64^*^	0.42	0.97	0.04
HF (ms)^*^	22	798.72	1476.86	22	575.77	945.5	22	660.24	818.73	22	407.35	335.59	0.91^*^	0.48	1.65	0.2
LF (nu)	22	48.5	16.60	22	52.86	15.85	22	53.02	16.35	22	43.91	14.46	−9.09	−18.48	0.30	<0.0001
HF (nu)	22	50.77	16.13	22	47.14	15.86	22	47.03	16.35	22	56.09	14.46	9.09	−0.30	18.48	<0.0001
LF/HF	22	1.35	1.01	22	1.67	1.12	22	1.57	1.07	22	1.0	0.64	−0.57	−1.11	−0.03	0.03
**INFLAMMATORY MARKERS**																
TNF (pg/ml)	22	12.22	4.82	22	12.49	4.68	22	12.79	4.41	22	10.30	4.02	−2.50	−5.06	0.07	0.03
Leptin (ng/ml)	22	27.90	14.93	22	33.08	21.91	22	33.48	17.18	22	27.91	19.34	−5.18	−16.97	5.81	0.001
Adiponectin (μg/ml)	22	8.76	1.75	22	8.72	1.85	22	7.04	1.51	22	9.35	1.70	2.30	1.32	3.28	<0.0001
Leptin/adiponectin ratio	22	3.32	1.86	22	4.03	2.51	22	4.96	2.78	22	3.12	2.09	−1.84	−3.38	−0.28	0.02

* *These variables were analyzed on the logarithmic scale*.

The pairwise difference (pre- to post-) of the measurements within each treatment arm, as well as the difference in the placebo group and the galantamine group at the final time point, were determined using Tukey *post-hoc* analysis. Unless otherwise specified, a result was considered statistically significant at the *P* ≤ 0.05 level of significance. All analyses were performed using SPSS version 20.0 (SAS Institute Inc., Cary, NC).

## Results

### Patients

**Figure 1:**
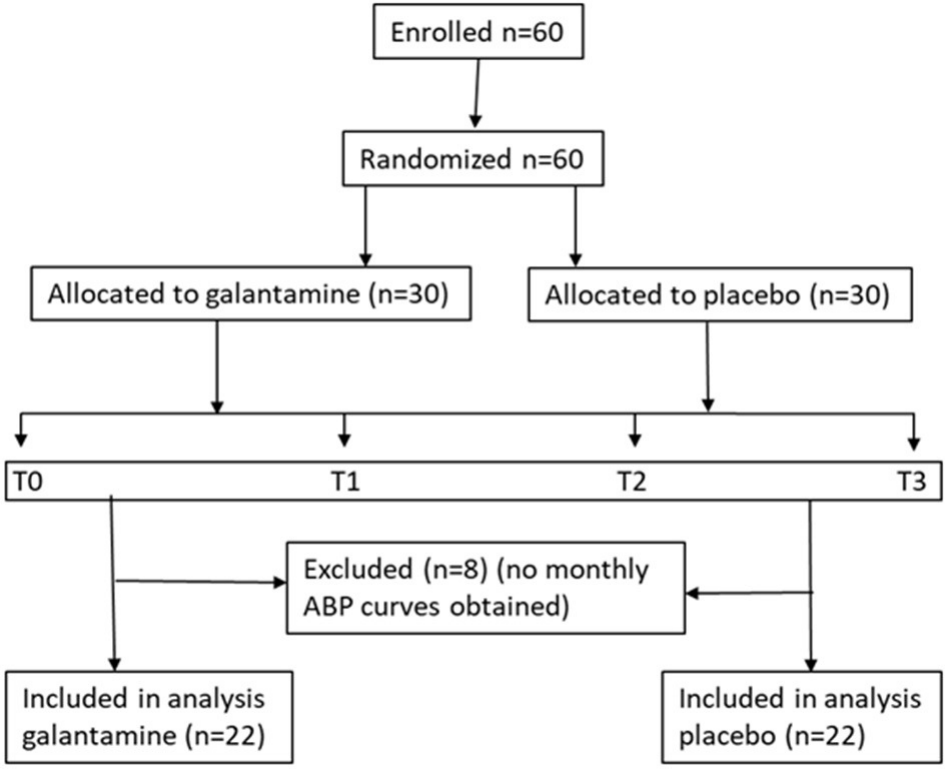
Flow chart (protocol sequence). T0—blood sample draw, blood pressure curve recording; T1 and T2—blood pressure curve recording; T3—blood sample draw, blood pressure curve recording.

Between March 2013 and March 2015, 189 patients provided informed consent. After initial screening, and consideration of the inclusion and exclusion criteria, 60 eligible subjects (30 males and 30 females) were consecutively randomized in a 1:1 ratio to treatment with galantamine and placebo (Supplementary Figure 1). Of the 60 patients enrolled in the original trial, 44 (22 in each arm) had complete HRV recordings available for analysis and were included in the current study (see Figure 1, trial profile). Male: female ratios were 12/10 in the placebo group and 9/13 in the galantamine group. There were no statistically significant differences between the placebo and galantamine groups in the baseline values of all variables analyzed, including age and anthropometry, metabolic, hemodynamic, inflammatory, and oxidative stress indices (Table 1). It should be noted that some of the SOD values were below the lower detection limits of the assay and these values were omitted from the study. Therefore, the analysis of SOD levels were performed based on data from 16 subjects in the placebo group and 12 subjects in the galantamine group. Of note, galantamine treatment was well-tolerated and as previously reported (35) only three adverse events were reported in both groups and categorized as minor based on the Common Terminology Criteria for Adverse Events (CTCAE 4.03, 2010): mild (grade 1) or moderate intensity (grade 2). These adverse effects were of short duration and no hospitalization was necessary. In the placebo group one subject had a single episode of nausea and diarrhea (grade 1) and another subject had a skin infection (grade 2). Of note, the skin infection was mild, occurred 7 weeks prior to the post treatment assessment, and was resolved within 3 days. Therefore, it would not have impacted the endpoints, including cytokine levels. In the galantamine group one subject complained of dizziness (grade 1).

### Effects of Galantamine on Oxidative and Nitrosative Stress Indices

Administration of galantamine conferred significant protection against oxidative stress. At the end of the 12-weeks treatment period, the activities of antioxidant enzymes, including SOD and CAT increased significantly in the galantamine treated subjects compared with the placebo group [+1.65 USOD/mg protein, [95% CI 0.39–2.92], *P* = 0.004] and [+0.93 nmol/mg, [95% CI 0.34–1.51], *P* = 0.01], respectively (Table 2). No significant effect of galantamine treatment was detected on GPx activity (−1.64 nmol/min/mg prot, [95% CI −9.31–6.03], *P* = 0.3] (Table 2). Galantamine decreased lipid peroxidation compared with placebo, as indicated by reduced levels of TBARS (a marker of oxidative damage [log scale 0.72 pmol/mg, [95% CI 0.46–1.07], *P* = 0.05] and systemic nitrite levels [log scale 0.83 nit/mg protein, [95% CI 0.57–1.20], *P* = 0.04] (Table 2). Galantamine treatment did not have a significant effect, compared with placebo, on protein peroxidation (determined by reaction of protein carbonyl groups) [−0.07 nmol/mg prot [95% CI −0.21–0.06], *P* = 0.2] (Table 2). However, a significant reduction in systemic nitrite levels was observed in subjects treated with galantamine compared with those treated with placebo [−0.05 μmol/mg prot, [95% CI −0.21–0.10], *P* = 0.04] (Table 2).

### Effects of Galantamine on Inflammatory and Metabolic Indices

We reanalyzed plasma samples for inflammatory and metabolic indices to address the important question of whether galantamine effects on oxidative stress were associated with retaining beneficial anti-inflammatory and metabolic effects of galantamine (35) in the 22 patients per group. Adiponectin levels were significantly increased [+2.30 μg/ml [95% CI 1.32–3.28], *P* < 0.0001] in the galantamine-treated patients compared with the placebo-treated (Table 3). In contrast, plasma TNF levels [−2.50 pg/ml [95% CI −5.06–0.07], *P* = 0.03], and leptin levels [−5.18 ng/ml [95% CI −16.97–5.18], *P* = 0.001], and leptin/adiponectin ratio [−1.84 [95% CI −3.38 to −0.28], *P* = 0.02] were significantly decreased in patients treated with galantamine compared with the placebo-treated) (Table 3). In addition, galantamine treatment significantly decreased plasma insulin levels and HOMA-IR compared with placebo (*P* = 0.01 for both effects) (Table 3). As previously reported (35) no significant differences between the groups were determined in BMI, waist circumference, HDL cholesterol levels, LDL cholesterol, triglyceride, and total cholesterol levels, systolic BP (SBP), diastolic BP (DBP), and heart rate (Table 3).

### Effects of Galantamine on Heart Rate Variability

**Figure 2:**
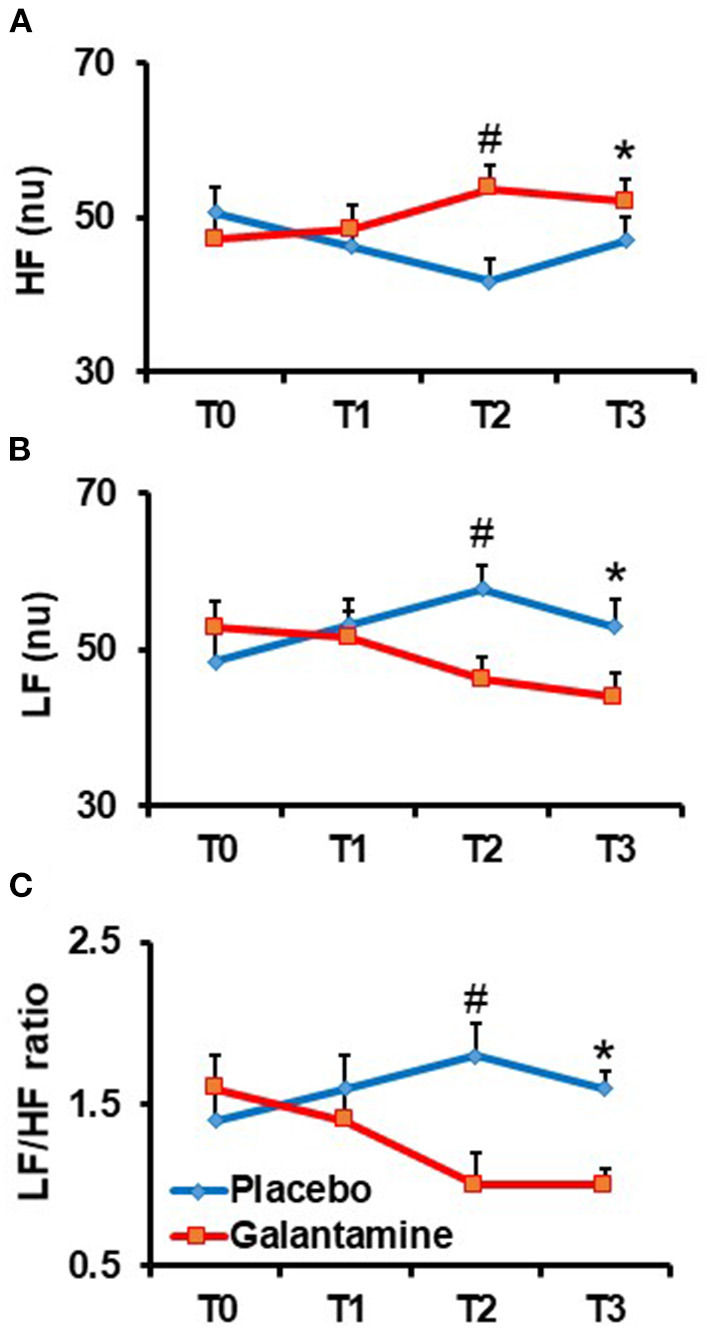
Time course of galantamine effect on heart rate variability (HRV) parameters in the frequency domain. Analysis of galantamine effects as compared to placebo was performed at randomization (T0), and after 4 (T1), 8 (T2), and 12 weeks (T3) of follow up. (A) Effect of galantamine on the high frequency (HF) component of HRV: ^#^*P* = 0.007 (T2 vs. T0); *P = 0.04 (T3 vs. T0). **(B)** Effect of galantamine on the low frequency (LF) component of HRV: ^#^*P* = 0.009 (T2 vs. T0); *P = 0.005 (T3 vs. T0) **(C)** Effect of galantamine on the LF/HF ratio: ^#^*P* = 0.001 (T2 vs. T0); *P* = 0.001 (T3 vs. T0).

We have previously reported that at the end of the 12-weeks treatment period there were significant changes in HRV parameters of MetS subjects treated with galantamine, compared with the placebo group, reflecting altered autonomic modulation (35). In this study, we found that these galantamine effects were preserved in the smaller cohorts (*n* = 22) of MetS patients (Table 3). The low frequency (LF) power (ms^2^) of HRV in the galantamine group was significantly decreased, compared with the placebo group [log scale 0.64 ms^2^ [95% CI 0.42–0.97], *P* = 0.04] (Table 3). The LF/HF ratio was significantly decreased in the galantamine group compared with the placebo group (*P* < 0.0001) reflecting the lower LF [normalized units [nu]] and the corresponding increase in high frequency (HF) (nu) (Table 3). Considering the importance of autonomic modulation in physiology and pathophysiological conditions (47), we further examined the time course of these drug effects on HRV in MetS subjects. Sequential analysis of HRV at T1 showed unaltered LF and HF (nu) after 4 weeks of galantamine (8 mg/days) treatment, compared with placebo (Figures 2A,B). Increasing the drug dose to 16 mg/days for 4 weeks resulted in significantly higher HF and lower LF at T2 (*P* = 0.007 and *P* = 0.009, respectively) (Figures 2A,B). These alterations were retained at T3 (end of study), after an additional 4 weeks of 16 mg/days galantamine treatment, compared with placebo (*P* = 0.04 and *P* = 0.005, respectively) (Figures 2A,B). Accordingly, the LF/HF ratio, a proposed index of sympatho-vagal balance was significantly decreased at T2 (*P* = 0.001) and T3 (*P* = 0.001) compared with placebo (Figure 2C).

## Discussion

To our knowledge, this is the first study to assess the effects of galantamine, a clinically approved cholinergic drug for the treatment of Alzheimer's disease on oxidative stress in patients with MetS. The results of the study demonstrate that within 12 weeks, relatively low doses galantamine significantly alleviate oxidative stress in parallel with amelioration of inflammation and insulin resistance, and beneficial alterations in autonomic regulation in MetS patients. These beneficial effects cannot be attributed to lowering BMI and waist circumference following drug treatment, because galantamine treatment did not significantly alter these indices.

Although the pathogenesis of MetS remains incompletely understood, oxidative stress, chronic inflammation, and autonomic dysfunction are thought to be key constituents that can be therapeutically targeted (2, 6, 11, 12, 17, 37). Intriguingly, it has been shown that even achieving weight loss through caloric restrictions is not necessarily associated with decreased levels of TNF and increased levels of adiponectin—molecules with an essential role in mediating the inflammatory state in MetS (48). These observations emphasize the need for targeted interventions directed toward ameliorating inflammation and the closely related oxidative stress—major drivers of insulin resistance and other metabolic derangements in MetS to decrease the risk of type 2 diabetes and cardiovascular disease. Impaired redox homeostasis and increased oxidative stress in MetS are directly linked to activation of pro-inflammatory and pro-thrombotic signaling that promote insulin resistance and suppress endothelial-mediated vasorelaxation, which results in cardio-metabolic complications (37). Considering the central role of oxidative stress as a driver of pathology in MetS (4, 5, 15, 37), in this study we focused on evaluating the effects of galantamine on oxidative stress indices. Galantamine, administrated for 12 weeks significantly increased the antioxidant SOD and CAT activities and did not alter GPx activity. Lipid peroxidation determined by TBARS, another indicator of oxidative damage, was significantly decreased following galantamine treatment. Galantamine also significantly decreased plasma nitrite levels. Previously, the effects of other modalities, including Mediterranean diet, melatonin, resveratrol, glycine, and vitamin E on oxidative stress in MetS have been studied (49–53). These treatments have reportedly variable effects on oxidative stress indices; some result in increased levels of SOD (49–51) while others cause a decrease in SOD-specific activity (52, 53) in parallel with affecting other indices of oxidative stress, including CAT, GPx, and malondialdehyde. However, no significant alterations in lipid and protein oxidative damage have been previously associated with these modalities. Our results, demonstrating the effects of galantamine on multiple components of oxidative and nitrosative stress in MetS patients provide novel insights extending the scope of beneficial efficacy of this clinically-approved drug.

In MetS, oxidative stress is interrelated with low-grade systemic inflammation and metabolic derangements, including insulin resistance (37). In this respect our results reveal galantamine anti-inflammatory effects in the cohort of 22 MetS patients in parallel with the beneficial drug effects on oxidative stress. Galantamine significantly inhibited TNF and leptin—two key mediators of the chronic inflammatory state and contributors to insulin resistance (10, 54, 55). Galantamine also increased levels of adiponectin—a molecule with anti-inflammatory properties, inversely associated with insulin resistance (10, 54, 55). The lower levels of insulin and insulin resistance in these patients indicated and strengthen the causative relationship.

In animal models, galantamine anti-inflammatory effects have been linked to brain muscarinic acetylcholine receptor-mediated activation of the vagus-nerve based inflammatory reflex (22, 23, 29, 32, 56, 57) and this drug has been shown to alter HRV and stimulate efferent vagus nerve activity (32, 34). Galantamine also alleviates hypertension in an animal model of lupus erythematosus (32). In addition, galantamine treatment or vagus nerve stimulation results in decreased inflammation in animal models of many diseases, including inflammatory bowel disease and arthritis with recent success in treating human conditions (23, 29, 33, 56, 58–62). In addition to vagus nerve cholinergic signaling, sympathetic catecholaminergic outflow has been linked to modulation of inflammation (20). In many conditions, including obesity-associated disorders, aberrant inflammation coexists with autonomic dysfunction (10, 63). HRV analysis provides a non-invasive approach to examine autonomic regulation of the heart and some, although limited insights into sympatho-vagal balance (44). Autonomic dysfunction manifested by increased LF and decreased HF, alongside inflammation and oxidative stress have been reported in MetS (10, 12, 13, 17). Autonomic dysfunction has been also strongly associated with morbidity and mortality in MetS, and cardiovascular disease (8, 10, 14). Accordingly, improving HRV and lowering the LF/HF ratio have been proposed as a therapeutic approach (8, 10, 12). HRV results from the current study indicate the effect of galantamine on measures of autonomic neural modulation, as reflected by lower LF/HF ratios. This alterations were already significant at the 8 week (T2) time point, e.g., after the first 4 weeks of galantamine 16 mg/days treatment. While somewhat speculative, considering the relatively small sample size of the study, it can be suggested that galantamine effects on autonomic regulation could contribute to anti-inflammatory and insulin resistance–alleviating effects of this drug.

Treatments with AChE inhibitors, including galantamine have been shown to reduce the risk of acute myocardial infarction and death in a nationwide cohort of subjects diagnosed with Alzheimer's dementia (64). Another study corroborates this information (65). A recent meta-analysis-based study highlights that galantamine and other AChE inhibitor treatments are associated with lower risk of CV events including stroke, acute coronary syndrome, and cardiovascular mortality (66). In addition, a very recent study reported that AChE inhibitor treatments are associated with lower mortality in patients with diabetes mellitus with Alzheimer's disease or patients with mixed-pathology dementia, with a specific benefit provided by galantamine or another AChE inhibitor—donepezil (67). In light of all of these findings, it seems reasonable to suggest that further studies with galantamine treatments would be of specific interest for patients with Alzheimer's disease with MetS or diabetes (68–70).

It should be noted that the beneficial effects of galantamine in MetS patients were observed using relatively low doses. The highest dose used (16 mg/days) in this study is less than the highest dose-−24 mg/days, approved for the treatment of AD patients with typically much lower BMIs. The study subjects tolerated well this dosage and none of them reported adverse effects. While galantamine significantly altered autonomic modulation (based on HRV analysis), no significant alterations were observed in heart rate and office BP. These findings are in line with a previous report in Alzheimer's patients treated with galantamine (68). The absence of adverse effects on cardiovascular and metabolic parameters indicate a good safety profile of galantamine treatment at the doses used. As this relatively low galantamine dose for a relatively short treatment time did not significantly alter lipid and glucose levels, and other parameters, future studies will provide insight into whether longer duration of galantamine treatment and/or higher drug doses result in a broader scope of beneficial alterations. In this context it would be of interest to study the effects of galantamine treatment on indices of hepatic pathology in patients with MetS as suppression of ALT and AST levels and amelioration of hepatic steatosis by this drug have these demonstrated in animal studies (30, 69).

This study has strengths and limitations. We have used parallel groups with both genders represented per group, concealed randomization, placebo control, and blinding of participants and investigators to meet the requirements of a good trial design. The effects of galantamine were assessed using well-validated tests and assays. A failure in performing complete HRV assessment in 8 patients per group (who did not come for this test) resulted in a lower number of patients included in the study, which represents a limitation of the study. In addition, no diet restrictions were implemented due to potential difficulties to sustain a certain uniform diet for the entire study. Therefore, diet could have affected some of the outcomes, which alongside not measuring HbA1C can also be considered as limitations of the study.

In conclusion, our results demonstrate that treatment with relatively low doses galantamine, a cholinergic drug in clinical use for the symptomatic treatment of Alzheimer's disease, alleviates oxidative stress in patients with MetS. They also confirm the beneficial drug effects on inflammation and insulin resistance, and provide a specific insight into the drug effects on autonomic modulation in these patients. These results, alongside previously published findings considerably strengthen the rationale for further studies with galantamine for therapeutic benefit in the growing at-risk population of people with MetS.

## Data Availability Statement

The raw data supporting the conclusions of this article will be made available by the authors, without undue reservation.

## Ethics Statement

The study protocol was reviewed and approved by the Institutional Review Committee and the Human Subject Protection Committee of the Heart Institute (InCor) and the Clinic Hospital (number 11672/555738), University of São Paulo. The patients/participants provided their written informed consent to participate in this study.

## Author Contributions

FC and VP designed the study. CS, KK, TL, AA, and KD acquired the data. CS and LM performed the statistical analyses. FC, CS, KD, MI, and LM interpreted the data. CS wrote the first draft of the report with inputs from FC and LM. FC, CS, LM, KK, KD, MI, TL, PO, VP, DB, and KT provided comments, participated in additional discussions, and revised the paper. VP, DB, PO, KT, and FC edited the text and finalized the manuscript. All authors approved the final version.

## Conflict of Interest

VP and KT have published patents (US 8,865,641 B2 and US 8,003,632 B2) broadly relevant to this work and have assigned their rights to the Feinstein Institutes for Medical Research. The remaining authors declare that the research was conducted in the absence of any commercial or financial relationships that could be construed as a potential conflict of interest.
