# Supplementary figures and images for: The Cholinergic Drug Galantamine Alleviates Oxidative Stress Alongside Anti-inflammatory and Cardio-Metabolic Effects in Subjects With the Metabolic Syndrome in a Randomized Trial
Source: Front Immunol. 2021 Mar 11;12:613979. doi: 10.3389/fimmu.2021.613979 (PMC7991724; doi:10.3389/fimmu.2021.613979)

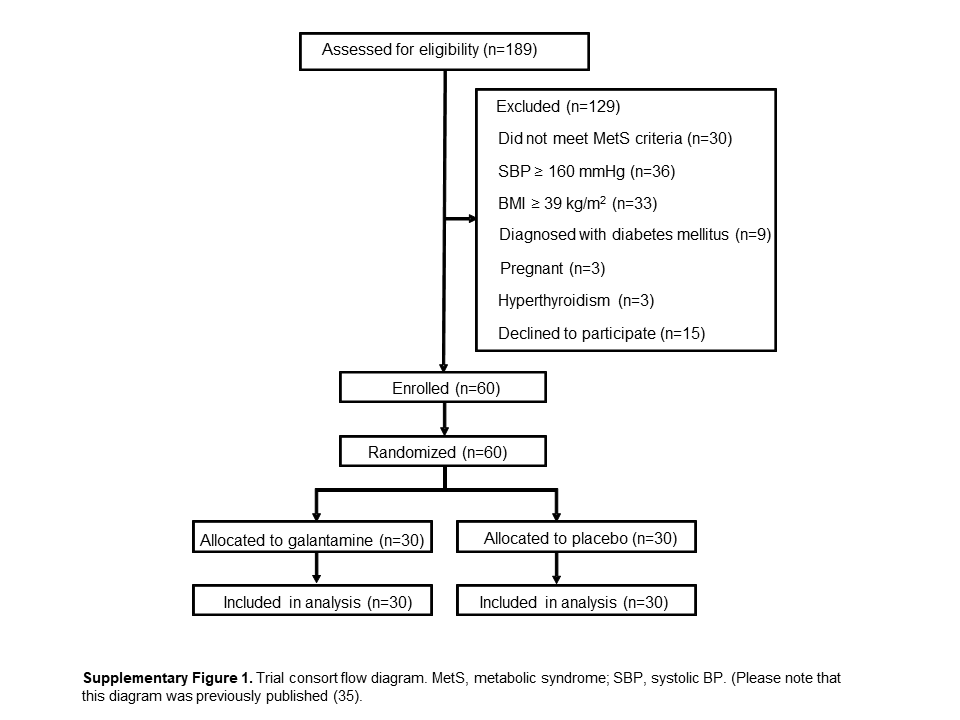

Supplement: Supplementary file 1 [file Image_1.TIF]
